# Research on the Relationships between Endogenous Biomarkers and Exogenous Toxic Substances of Acute Toxicity in *Radix Aconiti*

**DOI:** 10.3390/molecules21121623

**Published:** 2016-11-25

**Authors:** Haonan Zhou, Pengjie Zhang, Zhiguo Hou, Jiabin Xie, Yuming Wang, Bin Yang, Yanyan Xu, Yubo Li

**Affiliations:** Tianjin State Key Laboratory of Modern Chinese Medicine, School of Traditional Chinese Materia Medica, Tianjin University of Traditional Chinese Medicine, 312 Anshan West Road, Tianjin 300193, China; zhnwork09@163.com (H.Z.); 13752586596@163.com (P.Z.); hzgssh@163.com (Z.H.); 18600893359@163.com (J.X.); wangyuming226@163.com (Y.W.); yang3023008@163.com (B.Y.)

**Keywords:** *Radix Aconiti*, toxic substances, biomarkers, acute toxicity, corresponding relationships

## Abstract

*Radix Aconiti*, a classic traditional Chinese medicine (TCM), has been widely used throughout China for disease treatment due to its various pharmacological activities, such as anti-inflammatory, cardiotonic, and analgesic effects. However, improper use of *Radix Aconiti* often generated severe acute toxicity. Currently, research on the toxic substances of *Radix Aconiti* is not rare. In our previous study, acute toxic biomarkers of *Radix Aconiti* have been found. However, few studies were available to find the relationships between these endogenous biomarkers and exogenous toxic substances. Therefore, in this study, toxic substances of *Radix Aconiti* have been found using UPLC-Q-TOF-MS technology. Then, we used biochemical indicators as a bridge to find the relationships between biomarkers and toxic substances of *Radix Aconiti* through Pearson correlation analysis and canonical correlation analysis (CCA). Finally, the CCA results showed that LysoPC(22:5) is related to 14-acetyl-talatisamine, mesaconitine, talatisamine and deoxyaconitine in varying degrees; l-acetylcarnitine is negatively correlated with deoxyaconitine and demethyl-14-acetylkaracoline; shikimic acid has a good correlation with karacoline, demethyl-14-acetylkaracoline and deoxyaconitine; and valine is correlated with talatisamine and deoxyaconitine. Research on these relationships provides an innovative way to interpret the toxic mechanism of traditional Chinese medicine, and plays a positive role in the overall study of TCM toxicity.

## 1. Introduction

*Radix Aconiti* (Chuanwu in Chinese), the dried mother root of *Aconitum carmichaeli* Debx., has been widely used for thousands of years in clinical practice. As a famous and effective traditional Chinese medicine (TCM), it plays an important role in the treatment of diseases, such as rheumatoid joint pain, sciatica, paralysis and others [1]. However, the cardiotoxicity of this Chinese herbal medicine is severe, and the improper use of it will present highly toxicological risk and lead to fatal cardiac poisoning [2]. Aconitine, mesaconitine, hypaconitine and other diester-type alkaloids are known to be the main toxic substances of *Radix Aconiti* [3]. Modern research also showed that *Radix Aconiti* has an obvious toxicity caused by aconitine, and it usually produces abnormal heart function, fast respiratory rate, arrhythmia and other symptoms [4]. Generally, biochemical tests and histopathological observation are used to evaluate the safety, but these methods are time-consuming, have low sensitivity, are a heavy workload and have other shortcomings, thus we can not find toxicity during the early stage and explain mechanism of toxicity [5]. In the pre-experimental study, we found four acute toxic biomarkers of *Radix Aconiti*: shikimic acid, l-acetylcarnitine, LysoPC(22:5) and valine. Results showed that these endogenous biomarkers can quickly predict toxicity during the early stages [6]. However, the relationships between endogenous biomarkers and exogenous toxic substances are still not clear. This often hinders research on the toxic mechanism of TCMs. Therefore, there is an urgent need for a fast, accurate approach to find the toxic substances of TCMs, and study the correlations of endogenous biomarkers and exogenous toxic substances.

Metabolomics is a new approach developed following genomics, transcriptomics, and proteomics, and it is an important field of systematic biology [7]. Metabolomics could not only evaluate metabolism of biological samples, identify biomarkers, but also screen the active ingredients or toxic components in TCMs based on the overall efficacy [8]. As a new evaluation method, metabolomic biomarkers have been widely applied for efficacy assessment, toxicity evaluation and disease diagnosis with their unique advantages [9,10]. Li et al. has applied ten pre-determined biomarkers to evaluate whether the drugs have potential hepatotoxicity, and the results showed that these biomarkers are rapid, and have strong specificity, and high accuracy [11]. Liquid chromatography-mass spectrometry (LC-MS) is a detection methods that has high sensitivity and good accuracy, is easy operation and has other advantages [12,13]. In addition, using LC-MS for biological sample analysis in metabolomics studies, we could quickly and comprehensively obtain information of endogenous metabolites and plasma chemical compositions. Therefore, metabolomics technology and biomarkers were used in this experiment.

Generally, some serum substances of TCMs are the key factors in causing toxicity. The relationships between these exogenous substances and endogenous biomarkers can help explain the toxic mechanism of TCM. Therefore, this experiment was conducted to find relationships between endogenous biomarkers of *Radix Aconiti* and its exogenous substances. First, we identified toxic substances of *Radix Aconiti* in rat serum, and analyzed the change trends, followed by which we selected the toxic substances that varied linearly with the dose concentrations, and related to toxicity strength. These selected components were considered as potential toxic substances. Then we found out pre-determined biomarkers of *Radix Aconiti* by UPLC-Q-TOF-MS technology, and analyzed the variation trends. Finally, Canonical correlation analysis (CCA) was conducted to determine the correspondence between biomarkers and potential toxic substances. Research in this correspondence will help elaborate toxic mechanisms of TCM, and by this newly developed method we can quickly and accurately define the toxic substances in rat serum that really lead to toxic effects.

## 2. Results

### 2.1. Animal Behavior

After intragastric administration with different concentrations of *Radix Aconiti* ethanol extract, the animals suffered different degrees of abnormal motion. Drowsiness, ataxia, even convulsions, opisthotonos and other symptoms began to appear 30 min after administration. With the increase of administration concentration, these toxic effects gradually advanced. The toxic effect of high-dose group occurred early, obviously and severely. With the decrease of dosage, occurrence of toxicity grew less. However, the rats in normal saline group remained a normal state, and there was no abnormal behavior.

### 2.2. Biochemical Analysis

**Figure 1:**
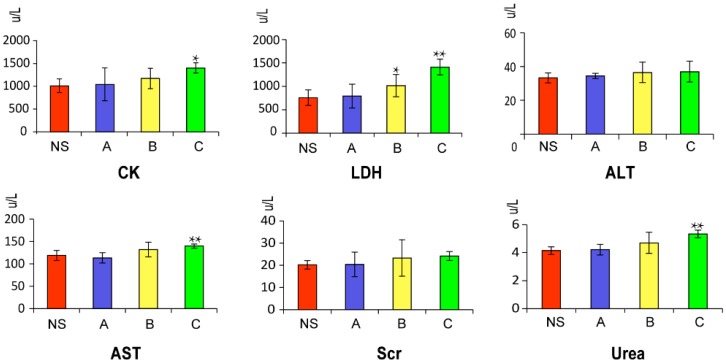
Effect of different concentrations of *Radix Aconiti* ethanol extract groups on various biochemical parameters in rats. Significant difference from control: * *p* < 0.05, ** *p* < 0.01. NS: Normal Saline; A: 2 g/kg ethanol extraction; B: 5 g/kg ethanol extraction; C: 10 g/kg ethanol extraction.

The detection and analysis of the enzymatic spectrum of serum are important to evaluate the extent of organ damage caused by toxic effects. In this study, we selected CK and LDH (heart damage detection index), ALT and AST (liver damage monitoring indicators), and Urea and Scr (kidney damage detection index) to evaluate the toxicity of *Radix Aconiti*. Creatine kinase (CK) and lactate dehydrogenase (LDH) are the commonly used indicators to assess the heart toxicity [14,15,16]. Their levels in the serum from the model group were compared with NS group by t-test. Kidney damage is indicated by increased contents of conventional indicators serum creatinine (Scr) and serum urea in the model groups compared with those in the control group [17,18]. Aspartate aminotransferase (AST), and alanine aminotransferase (ALT) values in the administration and normal control were compared to evaluate liver toxicity [19,20,21]. The biochemical results of our experiments are described in Figure 1. These biochemical parameters increased in a dose-dependent manner. Compared with control group, CK, AST, Urea and LDH significantly increased in the high dose (10 g/kg) group of *Radix Aconiti* extract; LDH also significantly increased in the middle dose group.

### 2.3. Histopathological Assessment

**Figure 2:**
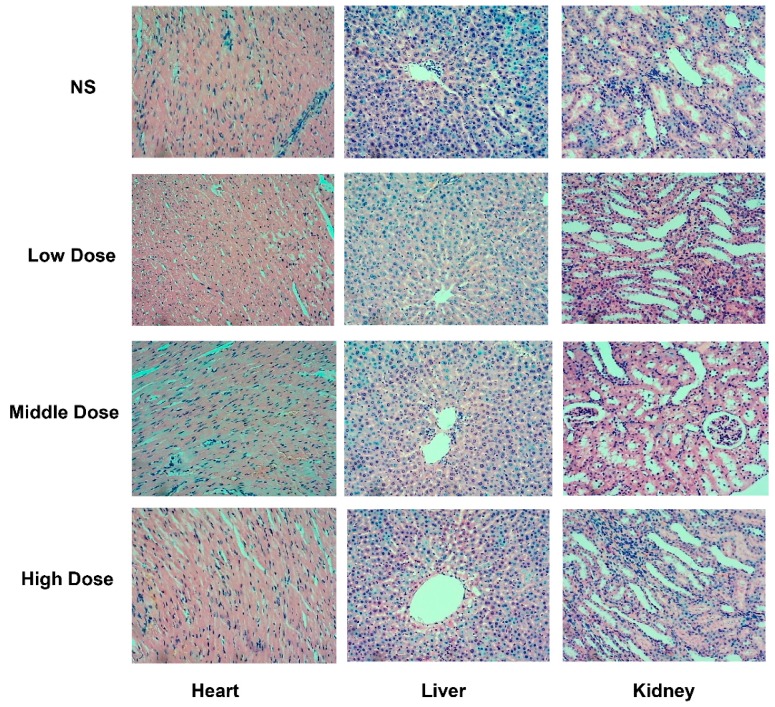
Pathological examination of heart, kidney and liver tissue after the administration of *Radix Aconiti* ethanol extraction. NS: Normal Saline; Low Dose: 2 g/kg ethanol extraction; Middle Dose: 5 g/kg ethanol extraction; High Dose: 10 g/kg ethanol extraction (10× magnification).

We used histopathological examination to evaluate the extent of tissue damage. The histopathological results are shown in Figure 2. In the NS group, normal basic structure of rat heart, liver and kidney tissue; arranged regular cardiac muscle fibers; clear structure of hepatic lobules; and normal number, morphology and distribution of glomerular in renal corticomedullary exist. In addition, there are no significant interstitial fibrosis in heart, liver and kidney tissue. In low dose group, part of the heart cell nuclear varies in size, mild edema around the central vein, part distal convoluted tubule and collecting duct atrophy with mild luminal expansion appear. In middle dose group, some dilated blood vessels under the heart tunica, blurred epithelial cavity of proximal tubule, slight edema around the central vein, and small pieces of scattered lymphocytes exist. In high dose group, heart cell nuclear mildly varies in size and within a slight shift, part of the central vein was dilated with slight edema in the surrounding, distal convoluted tubule and the collecting duct scatter epithelium atrophy with small tube expansion occur.

### 2.4. Pearson Analysis Results between Exogenous Toxic Substances and Biochemical Indicators for Acute Toxicity of Radix Aconiti

Typical UPLC-Q-TOF-MS spectrum of the rat serum and plasma fingerprint after intragastrically dosed with *Radix Aconiti* ethanol extraction is shown in Figures S1 and S2. The QC results of toxic substances and biomarkers met the requirement for testing exogenous and endogenous metabolites (shown in Tables S1 and S2). The basic peak ion (BPI) chromatograms of *Radix Aconiti* ethanol extraction are shown in Figure S3. According to the fingerprint spectrum, *m/z* values and MS^2^ information, we found the exogenous toxic substances which were both contained in rat serum and *Radix Aconiti* ethanol extraction. Finally, eight exogenous toxic substances were identified: karacoline, karacolidine, pengshenine A, demethyl-14-acetylkaracoline, talatisamine, 14-acetyl-talatisamine, mesaconitine and deoxyaconitine. Ion information of these toxic substances are shown in Table 1. The identification process of toxic substances is demonstrated in Supplementary Materials.

**Figure 3:**
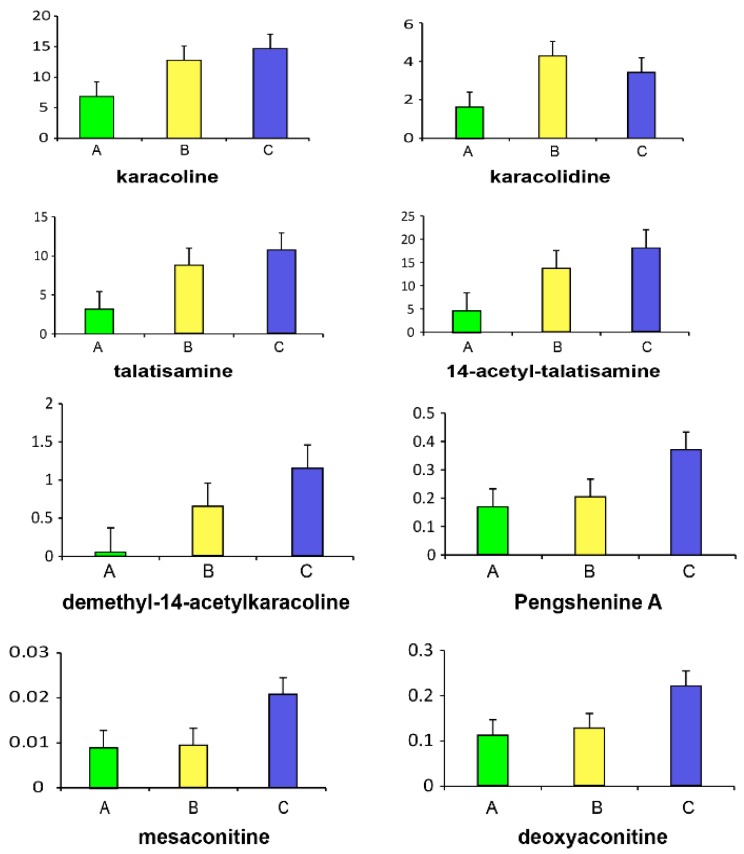
The variation trends of eight serum ingredients’ relative contents (peak area intensities) in different concentrations of *Radix Aconiti* ethanol extraction groups. A: 2 g/kg ethanol extraction; B: 5 g/kg ethanol extraction; C: 10 g/kg ethanol extraction.

We analyzed the variation trends of these toxic substances (shown in Figure 3). As shown in Figure 3, except karacolidine, the other seven toxic substances increased linearly with the increase of concentration. In addition, results of Pearson correlation analysis showed a good correlation between toxic substances and biochemical indicators except pengshenine A (Table 2). As the correlation coefficient approaches 1, the correlation between two variables is stronger. A positive value indicates a positive correlation, and a negative value indicates a negative correlation. Thus, it was indicated that these six toxic substances, which were dose-dependent and related to biochemical indicators, could be accurately considered as acute toxic substances of *Radix Aconiti*.

### 2.5. Correspondence between Endogenous Biomarkers and Exogenous Toxic Substances of Radix Aconiti

**Figure 4:**
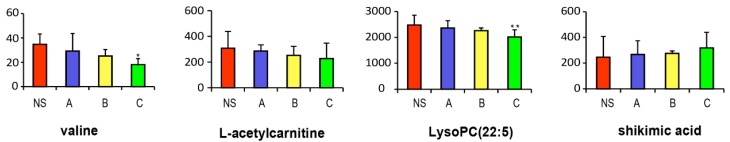
The variation trends of biomarkers’ relative contents (peak area intensities) in *Radix Aconiti* ethanol extraction groups with different doses. Significant difference from control: * *p* < 0.05, ** *p* < 0.01. NS: Normal Saline; A: 2 g/kg ethanol extraction; B: 5 g/kg ethanol extraction; C: 10 g/kg ethanol extraction.

Based on the previous study, we found four pre-determined biomarkers, shikimic acid, l-acetylcarnitine, LysoPC(22:5) and valine, and then we drew a histogram of content trends of these biomarkers (Figure 4). Simultaneously, we conducted Pearson correlation analysis between biomarkers and traditional biochemical indicators by SPSS 17 software. Results showed that these biomarkers seemed to be significantly dose-dependent and were obviously relevant to biochemical indicators (Table 3). The identification process of biomarkers is illustrated in Supplementary Material, and ion information of shikimic acid, l-acetylcarnitine, LysoPC(22:5) and valine are determined according to our previous study [6].

CCA was made to determine the correspondence between acute toxic dose-dependent biomarkers of *Radix Aconiti* and its toxic substances. During the CCA, we obtained four pairs of canonical variables, and the canonical correlation coefficients were 1.000, 0.904, 0.792, and 0.470. The closer the value of canonical correlation coefficient is to 1, the better correlation of endogenous biomarkers and exogenous toxic substances is. Normalization coefficients of biomarkers and toxic substances are shown in Table 4. Positive and negative coefficients reflect the trend between the indicators, and the absolute value of coefficient reflects the degree of correlation between the indicators. As seen in Table 4, LysoPC(22:5) was highly correlated with 14-acetyl-talatisamine and mesaconitine, and it also had a good correlation with talatisamine and deoxyaconitine. l-acetylcarnitine showed a good negative correlation with demethyl-14-acetylkaracoline and deoxyaconitine. Shikimic acid showed a high positive correlation with karacoline, and it is also related to demethyl-14-acetylkaracoline and deoxyaconitine. Valine was highly positively correlated with deoxyaconitine, and it also had a good negative correlation with talatisamine. All of the results indicated that there really are some relationships between endogenous biomarkers and exogenous toxic substances of *Radix Aconiti*. However, this needs further research to interpret how they affect each other.

## 3. Discussion

### 3.1. Potential Biological Significance of Exogenous Toxic Substances of Radix Aconiti on the Endogenous Biomarkers

In the results of CCA, LysoPC(22:5) has great correlation with mesaconitine, 14-acetyl-talatisamine, talatisamine and deoxyaconitine. LPCs, also called Lysophospholipids, are a type of endogenous phospholipid. It is a metabolite of PCs phospholipid, and PCs is an important component of biological membranes [6]. In addition, lysophospholipids can transmit lipid signal by means of lysophospholipid receptors (LPL-R). As a G-protein coupled receptor, LPL-R played a portion role of messenger in the organism life activities. [22,23] Some drugs can activate these receptors and send signals to activate lysophospholipids, which can affect the normal function of cardiomyocytes [9]. When cardiotoxicity was induced by traditional Chinese medicine or drug, the protein kinase C (PKC) pathway was activated, and phosphatase A2 activity was enhanced. This may lead to damage of the phospholipid membranes, and LPC reduction [24,25]. The over-activation of PKC may induce myocardial hypertrophy, apoptosis, and lead to structural and functional changes in cardiomyocytes, and, ultimately, affect cardiac function [26].

l-acetylcarnitine showed a good negative correlation with deoxyaconitine and demethyl-14-acetylkaracoline. l-acetylcarnitine is an esterified compound of l-carnitine, whose function is similar to l-carnitine. l-carnitine is a key substance of fat metabolism, and it could promote fatty acids into the mitochondria for oxidative decomposition [27,28]. Carnitines is thought to be the carrier for transporting fatty acid, and to increase the rate of fatty acids oxidation, reduce the consumption of glycogen [29]. Decrease of carnitines suggested that oxidation of myocardial fatty acid was inhibited, and fatty acid metabolism and sugars aerobic oxidation were reduced. These changes led to energy metabolic imbalance of myocardial cell, and eventually caused arrhythmias and myocardial damage [30].

*Radix Aconiti* (Chuanwu in Chinese) was already reported as a toxic Chinese herbal medicine because of the presence of diterpene alkaloids [31,32]. In this study, we eventually found six toxic substances: karacoline, demethyl-14-acetylkaracoline, talatisamine, 14-acetyl-talatisamine, mesaconitine and deoxyaconitine. Research has reported that mesaconitine has a greater toxicity on myocardial cells [33]. Weakness of muscules and death from respiratory depression can be induced by karacoline. Talatisamine may lead to brief hypotention and intestinal contractions [34].

### 3.2. Significance of Correspondence between Endogenous Biomarkers and Exogenous Toxic Substances of TCM

Traditional Chinese medicine contains complex chemical compositions. However, only a few individual substances might contribute to efficacy or toxicity. Thus, it is hard to find biologically active ingredients due to many interfering substances. In addition, it is also difficult to investigate pharmacological or toxicological mechanisms caused by various components. Therefore, we proposed a new method in this experiment to find toxic substances of TCM, and study the related mechanism that may occur. Using metabolomic technology and CCA, we found the correlation between endogenous biomarkers and exogenous toxic substances. These relationships had great significance for the research on the confirmation of toxic substances and toxic mechanism of *Radix Aconiti*. Meanwhile, this method provided an important reference for studying the mechanism of efficacy or toxic effect of TCM. In addition, the overlapping of multi-level analytical methods made the final obtained exogenous toxic substances more accurate. With the same batch of animals, studies of toxic substances and biomarkers for toxic evaluation could be conducted at the same time, which could greatly reduce the cost of research.

## 4. Experimental Section

### 4.1. Reagents and Materials

The acetonitrile and formic acid of high pressure liquid chromatography (HPLC)-grade were provided by Oceanpak (Gothenburg, Sweden) and ROE SCIENTIFIC INC. (Beijing, China), respectively. Distilled water was purchased from Wahaha (Hangzhou, China). Normal saline (NS) was obtained from Queensland Technology Co., Ltd. (Tianjin, China) [9]. *Radix Aconiti* was available from AnGuo, HeBei Province (Anguo City, China), and it was authenticated by professor Tianxiang Li (Tianjin University of Traditional Chinese Medicine, Tianjin, China). The contents of mesaconitine, aconitine, and hypaconitine in raw material are 0.2336 mg/g, 0.05805 mg/g, and 0.2932 mg/g, respectively (Detailed results of content determination were placed in Supplementary Material). 

### 4.2. Preparation of Radix Aconiti Extraction

*Radix Aconiti* was extracted by ethanol. Crushed herbs (50 g) were extracted with 10 times the amount of 70% ethanol by refluxed for 60 min to the residue of which 8 times the amount of 70% ethanol was added and refluxed for another 60 min. Then, the extracting solution was combined after filtrated, and was concentrated to 1 g/mL (amount of crude drug) 70% ethanol extract sample [6].

### 4.3. Experimental Animals and Groups

Forty male Wistar rats, weighing (200 ± 20) g, were supplied by the Academy of Military Medical Sciences experimental animal center (Beijing). The animal experiment was performed at the Institute of Radiation Medicine, Chinese Academy of Medical Sciences (Tianjin, China). The rats were housed in an SPF-level lab for one week adaptation, and the growth environment was as follows: 12 h day and 12 h night, ambient temperature of 23 ± 2 °C, ambient humidity of 35% ± 5%. The group, dose, administration mode and sampling time are shown in Table 5.

To minimize the suffering of animals, all experiments were performed in strict accordance with Chinese national laws and local guidelines. The animal study was approved by the Animal Ethics Committee of Tianjin University of Traditional Chinese Medicine with ethical approval number TCM-2012-078F01.

### 4.4. Sample Collection and Preparation

All animals were fasted for 12 h with access to water before sample collection. Each group was given ethanol extract of *Radix Aconiti* in corresponding concentration. Then, we took 8 mL of abdominal aorta blood at 30 min after dose. One milliliter of blood was placed in normal tubes and centrifuged at 3500 rpm for 10 min at 4 °C. The obtained supernatant was stored in a −80 °C freezer for screening toxic substances. Another 1 mL of the whole blood was handled and stored in the same conditions, and it was used for conventional biochemical test. Furthermore, we prepared another 1 mL of blood in heparinized tubes, then handled and stored it in the same method for metabolomics study. The remaining blood samples were placed in a −80 °C freezer for storage. After blood collection, we collected the heart, liver and kidney tissues from all rats, and immersed them in 10% formaldehyde solution. Then we observed pathological features of the tissues by haematoxylin and eosin (H&E) staining [6,11].

### 4.5. Chromatographic and Mass Spectrometric Conditions

In toxic substances study, prior to injection, 3 mL of acetonitrile was added to 1 mL of serum to precipitate the proteins, followed by centrifugation at 13,000 rpm for 10 min. The obtained supernatant was concentrated to dryness with nitrogen. Then, the residue was redissolved with 100 μL of aqueous methanol (1:1) and centrifuged again to obtain supernatant for analysis. In addition, 20 μL of *Radix Aconiti* ethanol extract was diluted by five times the amount of water, and then it was centrifuged at the same condition to obtain supernatant for analysis. Analysis was performed by a Waters UPLC-Q-TOF-MS system (Waters, Milford, MA, USA). All samples were randomly injected into ACQUITY UPLC HSS C_18_ column (2.1 mm × 100 mm, 1.7 μm, Waters) at 40 °C using 0.1% formic acid in water (A) and 0.1% formic acid in acetonitrile (B). The parameters were as follows: the flow rate was 0.3 mL/min, the injection volume was 5 µL and the gradient elution program utilized began with 90% A, then 90% A at 0–2 min, 90%–85% A at 2-7 min, 85%–70% A at 7–15 min, 70%–61% A at 15–21 min, 61%–50% A at 21–25 min, 50%–1% A at 25–26 min, 1%–1% A at 26–30 min, 1%–90% A at 30–30.5 min, and 90%–90% A at 30.5–40 min. The eluent was directly introduced to the mass spectrometer. To ensure the stability and repeatability of the systems, plasma samples singled out from each group were pooled as a quality control (QC) sample, which were injected and analyzed at an interval of 10 samples. For mass spectrometry, an electrospray ionization source (ESI) interface was used in positive mode. The MS parameters were fixed as follows: the electrospray capillary voltage was 3.5 kV, the drying gas flow was 10 mL/min, the gas temperature was 325 °C, the fragmentor voltage was 6 kV, the desolvation gas flow was 600 L/h, the nebulizer pressure was 350 psi, and the collision energy was 20–30 kV. Data were collected in the range of 50–1000 Da.

Metabolomic analysis was performed under the same conditions with a different gradient profiles. A linear gradient was used as follows: 99%–99% A at 0–0.5 min, 99%–50% A at 0.5–2 min, 50%–1% A at 2–9 min, 1%–1% A at 9–10 min, 1%–99% A at 10–10.5 min, and 99%–99% A at 10.5–12 min. The eluent was introduced to the mass spectrometer directly. Mass spectrometry analysis was performed on a Waters Q-TOF Premier with the same parameters of metabolomics. After the injection of 10 samples, a pooled sample, the QC sample, followed by a blank was injected in order to ensure the stability and repeatability of the systems [5,6].

### 4.6. Data Process

QC samples were used to validate the precision, reproducibility and stability of the specimen in LC-MS analysis. Twenty chromatographic peaks were randomly selected to calculate the relative standard deviations (RSD) of the peak areas and retention times to evaluate the precision and reproducibility. The RSD of the peak areas and retention times less than 15% indicated that the sample detection method meets metabolomics requirements. Data of toxic substances were analyzed using Masslynx (Waters) software 4.1. In the exported data, according to *m*/*z* values and corresponding MS^2^ information, we identified plasma substances of *Radix Aconiti* compared with the fingerprint of *Radix Aconiti* ethanol extraction. Then we analyzed the variation trends of these plasma substances (shown in Figure 3), and determined plasma substances which were dose-dependent. Subsequently, we found the relationships between plasma substances and biochemical indicators by Pearson correlation analysis. Finally, these plasma substances that showed a good correlation with biochemical indicators and had a dependent on dose were considered as toxic substances of *Radix Aconiti*.

Metabolomics original data were exported by using Masslynx (Waters) software. In the exported data, based on retention time, *m/z* values and corresponding MS^2^ information, we found pre-determined biomarkers, shikimic acid, l-acetylcarnitine, LysoPC(22:5) and valine, and then we drew a histogram of content trends of these biomarkers (Figure 4). Simultaneously, we conducted Pearson correlation analysis between biomarkers and traditional biochemical indicators by SPSS 17 software. Finally, we determined the dose-dependent biomarkers that were correlated to biochemical indicators.

### 4.7. Canonical Correlation Analysis

In general, research on the correlation between two sets of variables adopts simple correlation, linear correlation or regression analysis. However, these methods only consider a relationship between the individual evaluation index, and find it difficult to grasp the essence of the problem. CCA is a method of correlating linear relationships between two multidimensional variables. It can more comprehensively reflect the intrinsic relations between the variable groups, and is widely used to study the relationships between the variable groups [35]. In our experiment, exogenous toxic substances were set as X group and endogenous biomarkers were set as Y group to conducted CCA by using SPSS 17 software.

## 5. Conclusions

In this study, using biochemical indicators (CK, LDH, ALT, AST, Scr, and Urea) as the bridge, we developed a novel method to find the relationships between endogenous biomarkers and exogenous toxic substances of *Radix Aconiti* ethanol extraction by UPLC-Q-TOF-MS metabonomic method and CCA. First, we found eight toxic substances of *Radix Aconiti* and analyzed the correlation between biochemical indicators and these exogenous toxic substances. Then, we found four acute toxic biomarkers of *Radix Aconiti* based on the previous study, and analyzed the correlation between biochemical indicators and these endogenous biomarkers. Finally, we determined four biomarkers and six toxic substances of *Radix Aconiti*, which were dose-dependent and related to biochemical indicators. The relationships of these four biomarkers and six toxic substances were analyzed by means of CCA. Results showed that LysoPC(22:5) has different levels of correlation with mesaconitine, talatisamine, 14-acetyl-talatisamine and deoxyaconitine. l-acetylcarnitine showed a good negative correlation with demethyl-14-acetylkaracoline and deoxyaconitine. Shikimic acid was related to karacoline, demethyl-14-acetylkaracoline and deoxyaconitine. Valine was correlated with deoxyaconitine and talatisamine. This method provides a new idea for studying the toxic substances of TCM, and helps us quickly and accurately find toxic substances in rat serum that really lead to toxicity. At the same time, research on the relationships between endogenous biomarkers and exogenous toxic substances of TCM plays a positive role in revealing the complex toxic mechanisms of TCM in vivo.

## Figures and Tables

**Table 1:** Mass measurement for exogenous metabolites of *Radix Aconiti* ethanol extraction.

Metabolites	T_R_ (min)	*m/z*	Formula	MS/MS
Karacoline	1.77	378.2643	C_22_H_35_NO_4_	378.3, 360.2, 332.2, 328.2, 310.2
Karacolidine	1.82	394.2625	C_22_H_35_NO_5_	394.3, 376.2, 358.2, 344.2, 340.2, 326.2
Demethyl-14-acetylkaracoline	3.04	406.2597	C_23_H_35_NO_5_	406.3, 388.2, 356.2, 338.2
Talatisamine	6.05	422.2967	C_24_H_39_NO_5_	422.3, 390.3, 372.3, 358.2, 340.2
14-acetyltalatisamine	9.4	464.3051	C_26_H_41_NO_6_	464.3, 432.3, 400.3, 372.2
Pengshenine A	1.28	436.2327	C_24_H_37_NO_6_	436.2
Mesaconitine	16.82	632.3134	C_33_H_45_NO_11_	632.3, 600.3, 582.3, 572.3, 540.3
Deoxyaconitine	18.66	630.3338	C_34_H_47_NO_10_	630.3, 598.3, 570.3, 538.3, 506.3

**Table 2:** The correlation of biochemical indicators and exogenous components.

Metabolites	CK	LDH	ALT	AST	Scr	Urea
karacoline	0.858	0.843	0.986 *	0.810	0.916	0.888
karacolidine	0.709	0.692	0.950 *	0.761	0.876	0.763
demethyl-14-acetylkaracoline	0.987 *	0.985	0.942	0.966 *	0.979 *	0.997 **
pengshenine A	0.922	0.911	0.910	0.765	0.850	0.921
talatisamine	0.984 *	0.978 *	0.954 *	0.919	0.959 *	0.992 **
14-acetyl-talatisamine	0.990 *	0.985 *	0.939	0.908	0.944	0.993 **
mesaconitine	0.960 *	0.953 *	0.888	0.805	0.861	0.951 *
deoxyaconitine	0.967 *	0.958 *	0.950 *	0.865	0.925	0.972 *

*: Correlation is significant (*p* < 0.05) **: Correlation is highly significant (*p* < 0.01).

**Table 3:** The correlation of biochemical indicators and endogenous biomarkers.

Metabolites	CK	LDH	ALT	AST	Scr	Urea
valine	−0.963 *	−0.954 *	−0.949	−0.856	−0.920	−0.968 *
l-acetylcarnitine	−0.950 *	−0.942 *	−0.987 *	−0.907	−0.968 *	−0.970 *
LysoPC(22: 5)	−0.983 *	−0.978 *	−0.915	−0.866	−0.908	−0.980 *
shikimic acid	0.974 *	0.969 *	0.881	0.828	0.870	0.964 *

*: Correlation is significant (*p*< 0.05).

**Table 4:** The normalization coefficients of acute toxic biomarkers and toxic substances.

Toxic Substances	Biomarkers			
	y1	y2	y3	y4
x1	0.403	−0.191	1.270	0.136
x2	−0.287	−0.636	0.658	−0.229
x3	−0.587	−0.223	0.224	−0.558
x4	−1.111	0.097	0.427	−0.309
x5	1.444	−0.258	−0.353	−0.138
x6	0.585	−0.555	−0.832	0.867

x1: karacoline; x2: demethyl-14-acetylkaracoline; x3: talatisamine; x4: 14-acetyl-talatisamine; x5: mesaconitine; x6: deoxyaconitine; y1: LysoPC(22: 5); y2: l-acetylcarnitine; y3: shikimic acid; y4: valine.

**Table 5:** Group, dose, administration mode, and sampling time of rats.

Group	Number	Dose	Administration Mode	Sampling Time
NS	10	10 mL/kg	Intragastrically, single-dose	30 min
High dose	10	10 g/kg	Intragastrically, single-dose	30 min
Middle dose	10	5 g/kg	Intragastrically, single-dose	30 min
Low dose	10	2 g/kg	Intragastrically, single-dose	30 min

## Supplementary Materials

Supplementary materials can be accessed at: http://www.mdpi.com/1420-3049/21/12/1623/s1.
