# Supplementary material for: Research on the Relationships between Endogenous Biomarkers and Exogenous Toxic Substances of Acute Toxicity in Radix Aconiti
Source: Molecules. 2016 Nov 25;21(12):1623. doi: 10.3390/molecules21121623 (PMC6273418; doi:10.3390/molecules21121623)
Supplement: Supplementary file 1 [file molecules-21-01623-s001.pdf]

Supplementary Materials: Research on the Relationships between Endogenous Biomarkers and Exogenous Toxic Substances of Acute Toxicity in *Radix Aconiti*

Haonan Zhou, Pengjie Zhang, Zhiguo Hou, Jiabin Xie, Yuming Wang, Bin Yang, Yanyan Xu and Yubo Li

Part 1. Examples of Blood Toxic Substances Identification

With the (t_R = 1.77 min, *m*/*z* 378.2643) as an example to explain the process of identification of compounds. In the HMDB database, we using *m*/*z* to search to get molecular formula of compounds may be $C_{22}H_{35}NO_4$. In addition, the mass spectra fragments of compounds, 360.2, 332.2, 328.2, 310.2 *m*/*z*, corresponding to the loss of -H2O, $-CH_3-CH_3O$, $-CH_3OH-H_2O$, $-CH_3OH-2H_2O$. According to the fragment information, eventually concluded that the compound was karacoline.

Part 2. Examples of Biomarkers Identification

Used one of the biomarkers (t_R = 6.75 min, *m*/*z* 570.3529) as an example to explain the process of identification of compounds. First, we using m/z to search to get molecular formula of compounds may be $C_{30}H_{52}NO_7P$ in the HMDB database. In addition, the mass spectra fragments of compounds, 552.3, 184.1 and 125.0 *m*/*z*, corresponding to the loss of $-H_2O$, $-C_{21}H_{41}NO_3P$, $-C_{28}H_{47}NO_3$. According to the fragment information, eventually concluded that the compound was LysoPC(22:5).

Table S1. The results of experimental methodology in toxic substances research.

Experiment Name	RSD (Retention Time)	RSD (Peak Area)
Precision instrument	<0.72%	<8.83%
Method repeatability	<1.0%	<13.11%
Sample stability	<0.97%	<14.88%

Table S2. The results of experimental methodology in metabolomics research.

Experiment Name	RSD (Retention Time)	RSD (Peak Area)
Precision instrument	<0.33%	<8.32%
Method repeatability	<0.92%	<14.2%
Sample stability	<0.97%	<14.76%

Table S3. The content determination results of aconitine, mesaconitine and hypaconitine in *Radix Aconiti.*

Aconitine		Mesaconitine		Hypaconitine	
Y_1	X_1	Y_2	X_2	Y_3	X_3
0.00546	35463	0.0091	114725	0.0316	314707
0.00728	75141	0.0364	513775	0.0632	667599
0.0091	104213	0.0546	745070	0.0948	1006481
0.0273	230553	0.0728	1006322	0.1264	1126213
0.0455	352007	0.091	1262329	0.316	3427584
0.0637	650468	—	—	—	—

Y_1: Concentration of aconitine (mg/ml); X_1: Peak area of aconitine; Y_2: Concentration of mesaconitine (mg/ml); X_2: Peak area of mesaconitine; Y_3: Concentration of hypaconitine (mg/ml); X_3: Peak area of hypaconitine.

Standard curve equation:

$Y_1 = 1.000 \times 10^7 X_1 - 0.9888 \times 10^4$ $R^2 = 0.9650$

$Y_2 = 1.000 \times 10^7 X_2 - 0.7161 \times 10^4$ $R^2 = 0.9996$

$Y_3 = 1.000 \times 10^7 X_3 - 0.7386 \times 10^5$ $R^2 = 0.9931$

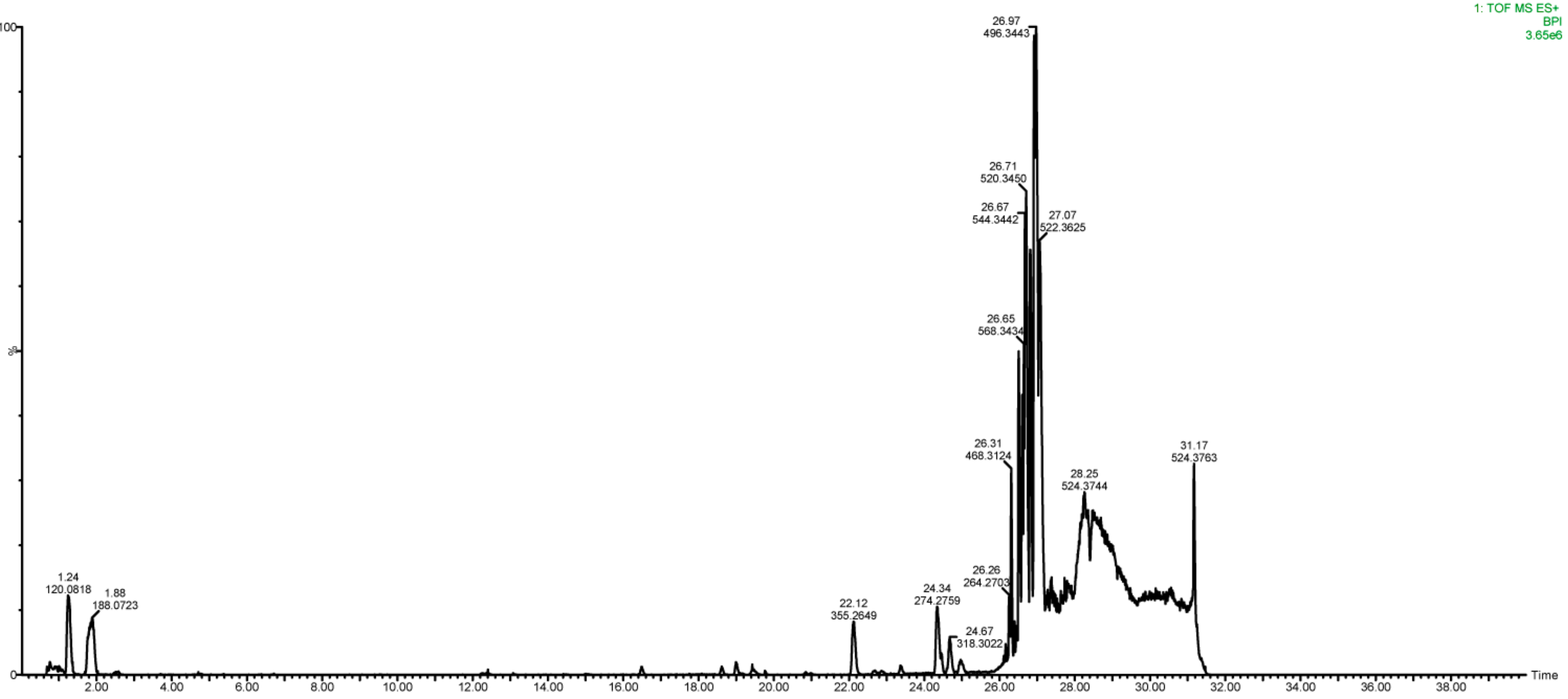

Figure S1. Basic peak ion (BPI) chromatograms of serum chemical substances metabolic profiling in rats.

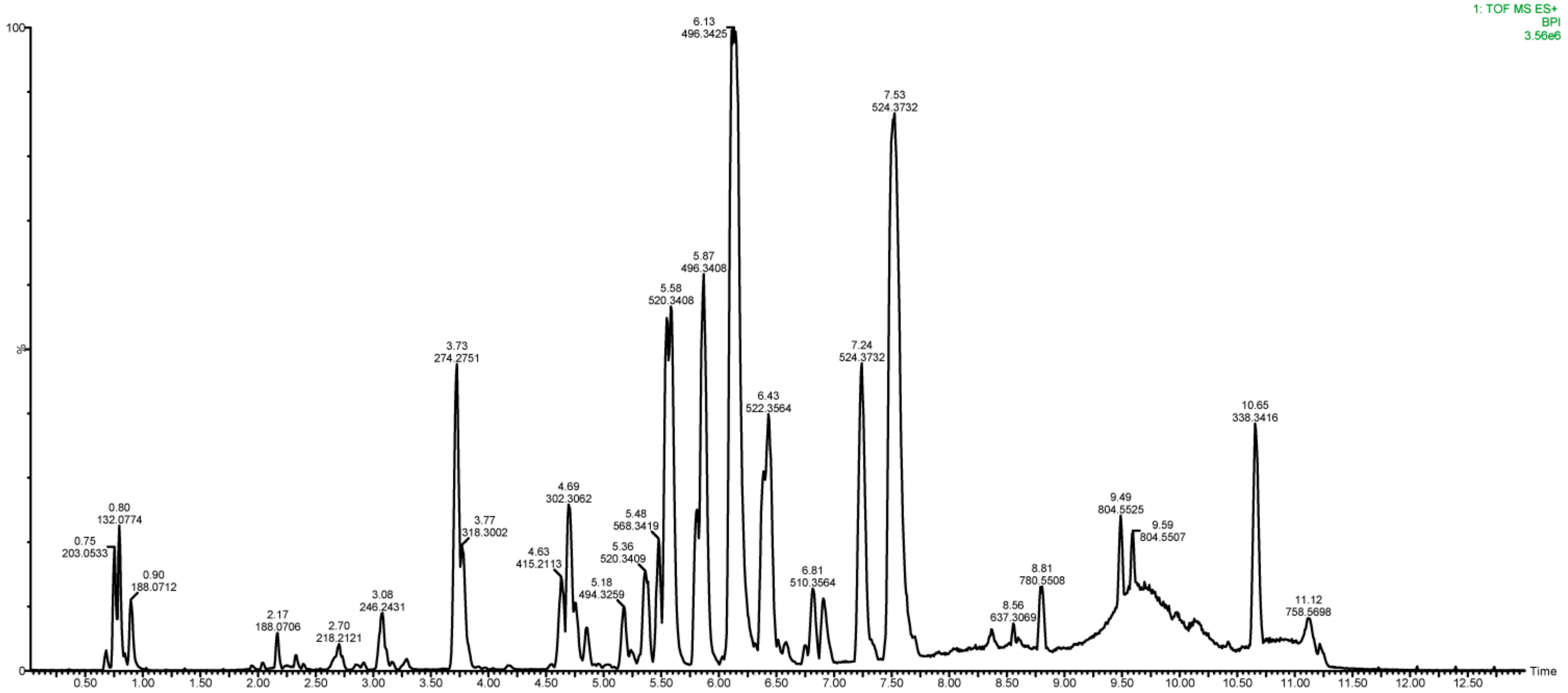

Figure S2. Basic peak ion (BPI) chromatograms of plasma metabolic profiling in rats.

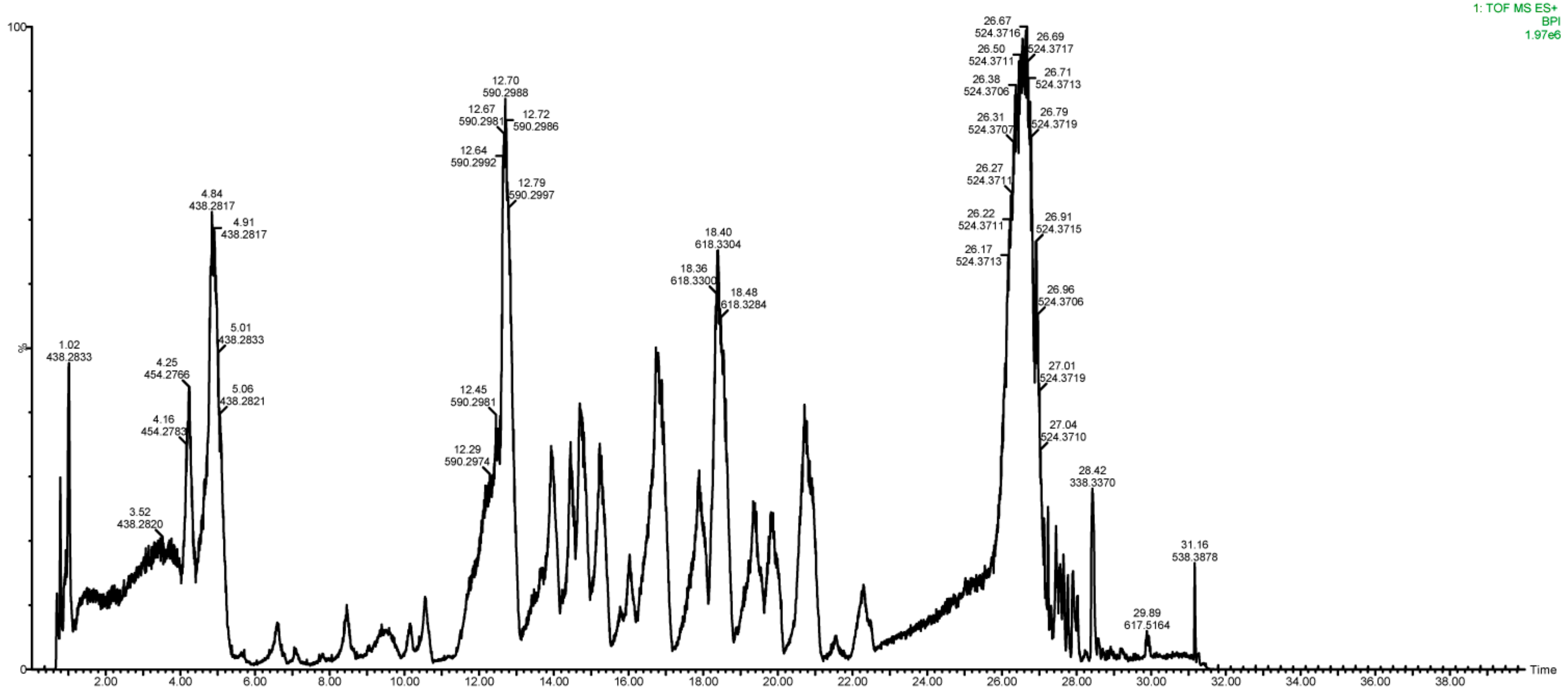

Figure S3. Basic peak ion (BPI) chromatograms of Radix Aconiti ethanol extraction.
